# Structure Elucidation and Toxicity Analysis of the Degradation Products of Deoxynivalenol by Gaseous Ozone

**DOI:** 10.3390/toxins11080474

**Published:** 2019-08-15

**Authors:** Mengmeng Li, Erqi Guan, Ke Bian

**Affiliations:** College of food science and technology; Henan Food Crop Collaborative Innovation Center, Henan University of Technology, Zhengzhou 450001, Henan, China

**Keywords:** deoxynivalenol, gaseous ozone, degradation products, toxicity

## Abstract

Fusarium Head Blight (FHB) or scab is a fungal disease of cereal grains. Wheat scab affects the yield and quality of wheat and produces mycotoxins such as deoxynivalenol (DON), which can seriously threaten human and animal health. In this study, gaseous ozone was used to degrade DON in wheat scab and the degradation products of ozonolysis were analyzed by ultra-performance liquid chromatography quadrupole-orbitrap mass spectrometry (UHPLC Q-Orbitrap). Toxicology analyses of the degradation products were also studied using structure-activity relationships. Ozone (8 mg L^−1^ concentration) was applied to 2 μg mL^−1^ of DON in ultrapure water, resulted in 95.68% degradation within 15 s. Ten ozonized products of DON in ultrapure water were analyzed and six main products (C_15_H_18_O_7_, C_15_H_18_O_9_, C_15_H_22_O_9_, C_15_H_20_O_10_, C_15_H_18_O_8,_ and C_15_H_20_O_9_) were analyzed at varying concentrations of ozone and DON. Structural formulae were assigned to fragmentation products generated by MS^2^ and Mass Frontier^®^ software. According to structure-activity relationship studies, the toxicities of the ozonized products were significantly decreased due to de-epoxidation and the attack of ozone at the C_9-10_ double bond in DON. Based on the results of the study above, we can find that gaseous ozone is an efficient and safe technology to degrade DON, and these results may provide a theoretical basis for the practical research of detoxifying DON in scabby wheat and other grains.

## 1. Introduction

Deoxynivalenol (DON) is a natural-occurring mycotoxin mainly produced by Fusarium graminearum that frequently infect cereal grains, especially wheat on the field or during storage [1]. Updated research indicated that DON is a major trichothecene in naturally infected grain and high toxicity in its by-products in Europe, China, and other major wheat producing areas [2,3,4]. DON affects the yield and quality of wheat and can seriously threaten human and animal health by causing acute temporary nausea, vomiting, diarrhea, headache, and fever [5]. In the consideration of wide prevalence of DON and its strong toxicity, lots of countries and regions have already established legislative limits for DON. The maximum permitted levels for DON is 1000 μg kg^−1^ in grains and their finished products in China [6], in Uniao Europeia (EU) legislation, is 1750 μg kg^−1^ in durum, 750 μg kg^−1^ in corn flour, and 200 μg kg^−1^ in infant food [7]. Based on the stringent legislation, the removal of DON from agricultural products, especially the scabby wheat, is an area of research interest [8,9,10]. 

Although a variety of chemical, physical, and biological treatments have been researched to reduce or eliminate DON in contaminated wheat [11,12,13], chemical treatment by ozone has been studied very little. This is probably due to the low diffusion of ozone and legislation prohibiting chemical treatment. At present, chemical treatment is forbidden in most countries for mycotoxins detoxification analyses due to uncertainty over the identity of the reaction products. However, ozone is gradually being used in the food industry due to its many advantages [7]. Ozone has been used as a fumigant to disinfest stored products and degrade mycotoxins in cereal grains [14,15]. The US Food and Drug Administration (FDA) declared O_3_ is safe for bottled water in the United States and can be used as a secondary direct food additive [16]. The ozonation of DON was reported to have encouraging degradation rates in several studies [17,18]. However, mechanisms of detoxification, structures, stability, and toxicity of ozonation products and potential side effects of the ozone detoxification have been poorly studied.

Ultra-high performance liquid chromatography tandem mass spectrometry (UHPLC-MS/MS), a cutting-edge molecule separation and determination technique, has been applied to the analysis of unknown compounds, especially degradation products of mycotoxins according to many published researches [19,20]. Q Exactive™ benchtop LC-MS/MS combining high-performance quadrupole precursor selection with high-resolution, accurate-mass (HR/AM) Orbitrap detection for outstanding performance and tremendous versatility, especially important in analyzing complex degradation products [21]. The Q Exactive™ mass spectrometer is superbly suited to untargeted or targeted screening with high-confidence confirmation, but is equally capable of a broad range of qualitative and quantitative applications. Moreover, MS^2^ provide complementary structural information through in-source fragmentation using high-energy collisions induced dissociation (HCD), which is crucial for structural analysis. In addition, data analysis software Mass Frontier that supplied by Thermo Scientific offers a confident path from spectra to structure through chemically intelligent structural elucidation, it integrates with Q-Orbitrap and provides an effective method for the analysis of complex degradation products and identification of the structure.

The objective of this study is to evaluate the efficacy of ozone treatment on DON level in ultrapure water, and to identify the degradation products of DON treated by gaseous ozone. In addition, to analyze the toxicity of ozonation products based on structure-activity relationship analysis, which will provide a theoretical basis for the practical research of detoxifying DON by gaseous ozone in scabby wheat from the aspect of food-safety. 

## 2. Results and Discussion

### 2.1. Effect of Gaseous Ozone on DON in Ultrapure Water

Data in Table 1 indicated that when the DON was dissolved in ultrapure water, with increasing of ozone concentration, the degradation efficiency of the DON increased regularly. When 8 mg L^−1^ gaseous ozone was treated with a 2 μg mL^−1^ of DON in ultrapure water, the degradation rate of DON was 95.68% within 15 s, which suggested that DON in ultrapure water was sensitive to gaseous ozone.

Ozone has strong oxidation, more free radicals are generated when the ozone concentration is higher, and free radicals like hydroxyl radical (·OH) can react with DON in the solution which lead to degrade DON. Generally, ozonolysis efficiency of mycotoxins increases with the increasing of ozone concentration [22]. When the cereal grains containing mycotoxins were treated by gaseous ozone, all kinds of influencing factors, such as ozone concentration, exposure time, DON contaminated way, and moisture of grain kernel must be considered for the sake of safety, nutrients, and sensory quality of cereal grains. We found that no significant detrimental changes in the protein content, pasting properties, and water absorption of the wheat samples were observed after treated with 90 mg L^−1^ gaseous ozone within 4 h. However, ozone concentration could not increase without limit due to the deteriorative effects on quality of treated samples.

### 2.2. Ozonation Products Analysis of DON

#### 2.2.1. Screening of Degradation Products

**Figure 1:**
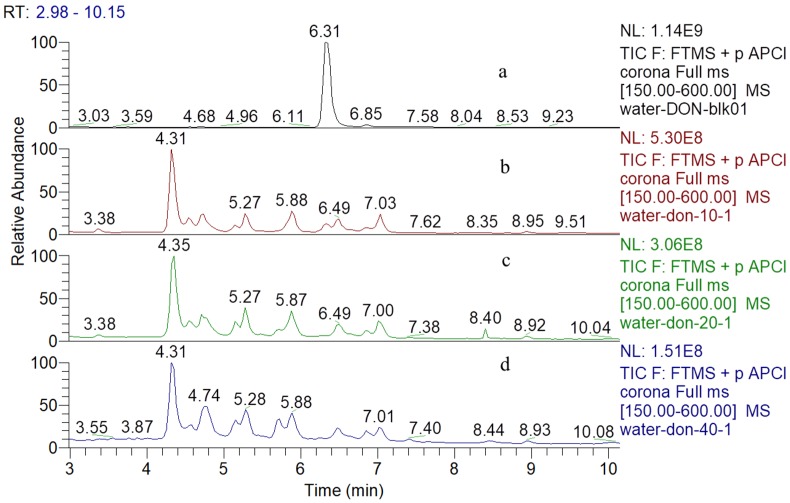
UHPLC-Q-Orbitrap MS total ion chromatograms corresponding to (**a**) DON in the ultrapure water before ozone treatment and (**b**–**d**) were ozonation products of DON in the ultrapure water after 10 mg L^−1^, 20 mg L^−1^ and 40 mg L^−1^ ozone treatment, respectively.

The total ion chromatograms (TIC) of DON and its degradation products which treated by various concentrations of gaseous ozone were shown in Figure 1. The retention time (RT) of DON was 6.31 min detected by UHPLC-Q-Orbitrap MS (atmospheric pressure chemical ionization source positive ionization mode), and 20 μg mL^−1^ DON was completely degraded after three concentrations of gaseous ozone treatment, most of the degradation products distributed from 4 min to 8 min, almost all of them were unknown compounds.

**Figure 2:**
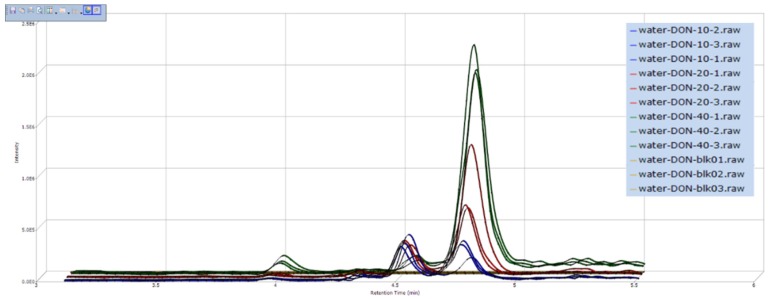
The result of degradation products screening by SIEVE 2.0. The abscissa represents retention time and ordinate represents intensity of degradation products. The three yellow lines that were parallel to abscissa represents DON in the ultrapure water before ozone treatment, and the icon was Water-DON-blk-01, 02, 03 (it means water-DON-blank-parallel sample 1, 2, 3). Similarly, the blue, red, and green lines represent DON in the ultrapure water after 10 mg L^−1^, 20 mg L^−1^, and 40 mg L^−1^ ozone treatment, respectively.

In our study, the screening of degradation products was performed using a differential expression analysis software SIEVE 2.0, which combined with Q-Orbitrap. After collected by Xcalibur 3.0 (data collection software of Q-Orbitrap), the original experimental data were imported to SIEVE 2.0, and the results were shown as the example in Figure 2. From Figure 2, the compound has chromatographic peak in the same RT and the intensity was different after detoxified by three concentrations of ozone, the higher ozone concentration, the higher ion intensity, so this unknown compound was the possible degradation product of DON. In addition, validation of the degradation products could be completed by comparing SIEVE figure with the RT and exact masses of the primary mass spectrogram. With this screening method, ten ozonation products of DON were analyzed and the information was listed in Table 2. 

In order to avoid missing the degradation products, the initial concentration of 100 μg mL^−1^, 50 μg mL^−1^, and 20 μg mL^−1^ of DON was degraded by 10, 20, and 40 mg L^−1^ gaseous ozone for 180 s, respectively. When all of these experimental levels were analyzed together, six major peaks were observed of each level. As shown in Table 3, six different degradation products could be speculated through retention time and the shape of each peak revealed the satisfactory separation effect.

#### 2.2.2. Structure Elucidation of Degradation Products 

**Figure 3:**
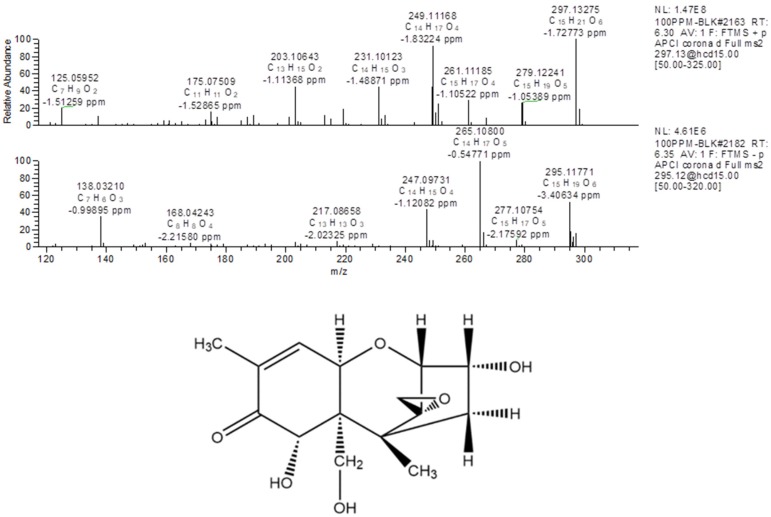
The MS/MS fragment ion information (±APCI) and structure of DON.

For structural elucidation of degradation products of DON treated by gaseous ozone, the data were analyzed using Q-Orbitrap MS with APCI. Figure 3 showed MS/MS fragment ion information (±APCI) and structure of DON standard. From the report of Young [18], the DON degradation began with attack of ozone at the C_9-10_ double bond with the net addition of two atoms of oxygen, so the basic structure of degradation products were the same as DON and C_9-10_ double bond was opened. In addition, the degree of unsaturation (the last column of Table 2), quantity of each degradation product that was a calculated indication for the number of double bonds and/or rings in the compound structure, were useful for structure speculation. DON is known to consist of 15 carbon atoms, 20 hydrogen atoms and 6 oxygen atoms, and the degree of unsaturation is 6. According to the structure of DON, the degree of unsaturation and the MS/MS fragment ion information of six key degradation products were listed in Table 4, the possible structure can be deduced.

**Figure 4:**
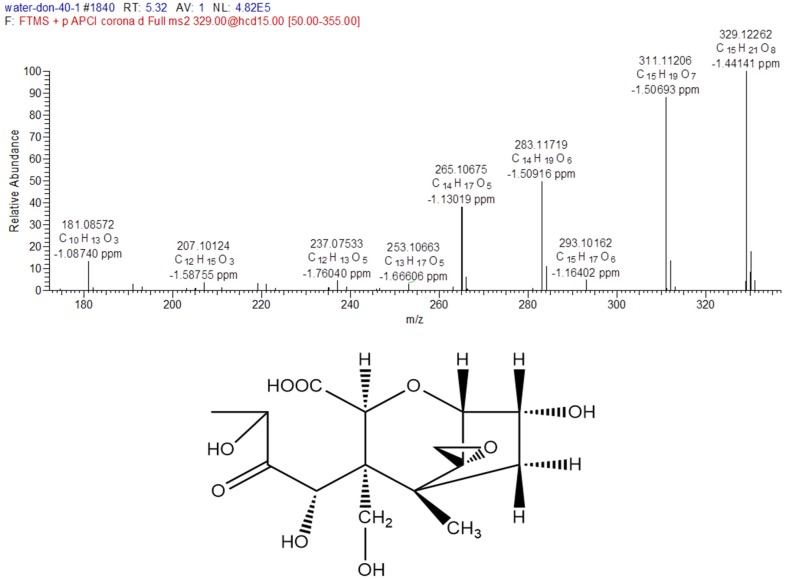
The MS/MS spectrum (+APCI) and possible structure of degradation product C of DON.

The MS/MS spectrum of degradation product C (Figure 4) was provided as an example to illustrate the analyzing process. The formula of degradation product C is C_15_H_22_O_9_, which were three oxygen atoms and two hydrogen atoms more than DON, and one degree of unsaturation lower than DON. The mass spectrum of product C showed a specific molecular base ion at *m*/*z* 329.12262 ([M+H-H_2_O]^+^), the key fragment ions were *m*/*z* 311.11206, which lost a molecule of H_2_O and *m*/*z* 283.11719, which lost a group of CO, and the fragment ions because of the fragmentation of ring. According to the reaction rule between ozone and DON, the ozone firstly attacked the C_9-10_ double bond, and then generated carboxyl through the addition reaction. Based on the above analysis and mass spectrometry fragmentation pathway, the possible structure of product C was shown in Figure 4.

#### 2.2.3. Fragmentation Pathway of the Ozonation Products

**Figure 5:**
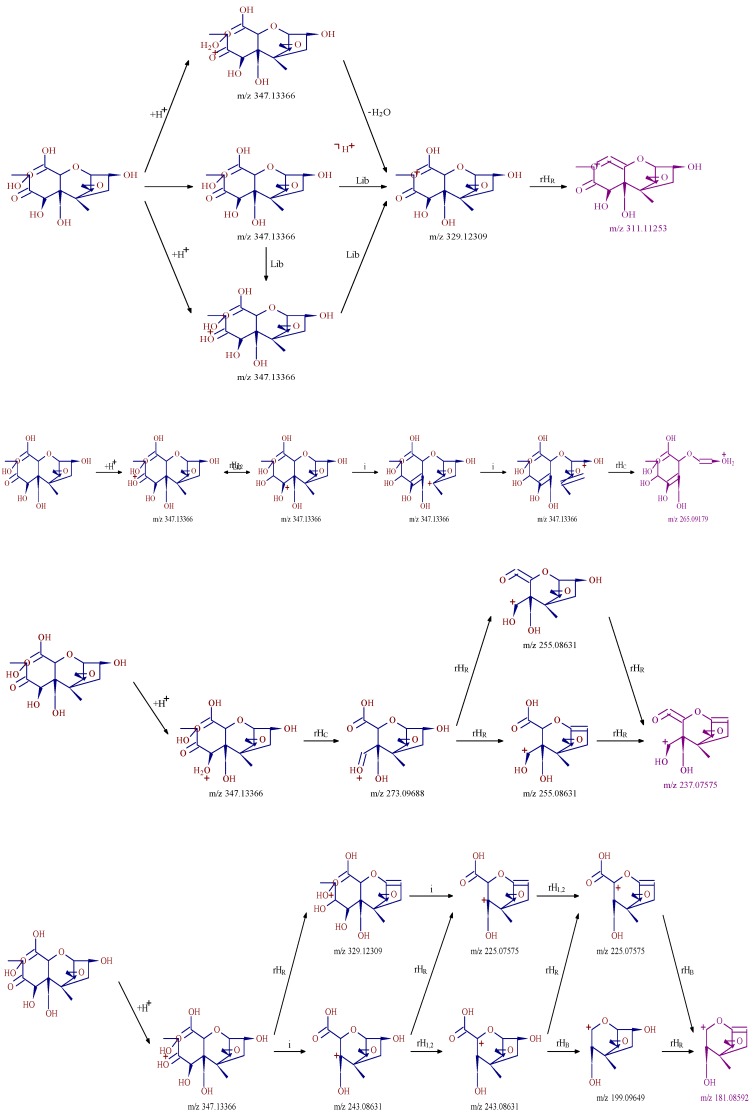
Fragmentation pathway of the ozonation product C (forming pathway of key ion fragments of product C).

**Figure 6:**
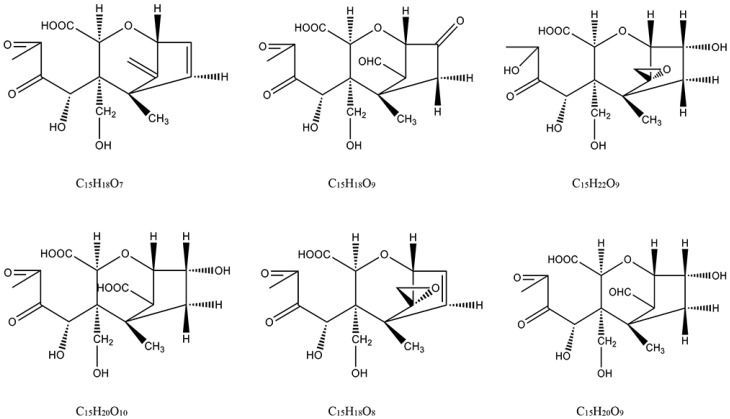
Structures of six key ozonation products of DON.

Mass Frontier 7.0 is a chemically intelligent structural elucidation software, which can elucidate the fragmentation pathway of degradation products. Importing the structures of degradation products into the software, the ion fragment and fragmentation pathway will generated base on the mass spectrometry fragment pathways database. The structure will be validated if this information was accorded with MS/MS fragment ion information. The fragmentation pathways of degradation product C were shown in Figure 5, the key ion fragments were *m*/*z* 311.11253, *m*/*z* 265.09179, *m*/*z* 237.07575 and *m*/*z* 181.08592, which were exactly consistent with the MS/MS fragment ion information in Figure 4, this indicated that the correct structure of product C in Figure 4 was obtained. Similarly, the other possible structures of ozonation products of DON can be proposed, the six key degradation products of DON were demonstrated in Figure 6.

### 2.3. Toxicity Analysis of Degradation Products

**Figure 7:**
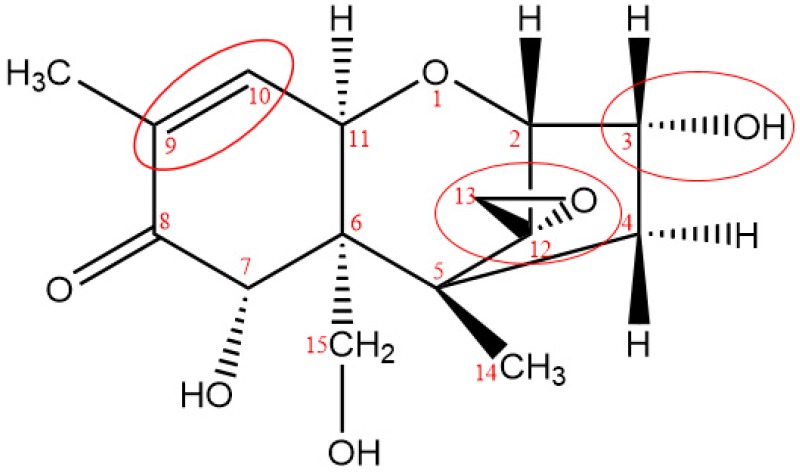
Key toxicity functional groups of DON.

Relationship between chemical structure and biological activities of many mycotoxins has been described in the literatures [23,24,25], all the results indicated that, toxicity of mycotoxin was closely related to functional groups of the molecular structure, which induced the theoretical basis for investigation of mycotoxin detoxification. The most important structural features that are known to affect toxicity activities of DON are as follows: the unsaturated bond at C_9-10_, the presence of the 12, 13-epoxy ring, and the presence of hydroxyl or other groups at appropriate positions [26], as shown in Figure 7.

The 12, 13-epoxide ring is responsible for the toxicity of DON [27], the hydrogenated opening of the 12, 13-epoxide ring results in the non-toxic compound [28]. From the report of Eriksen [29], the toxicity of DON expressed as the concentration inhibiting 50% of the DNA synthesis (IC_50_); the results indicated that the IC_50_ value for de-epoxy DON was 54 times higher in the assay than the IC_50_ for DON, and the results verified previous statement that the de-epoxidation is a detoxification reaction. As reported by Young [18], DON degradation began with attack of ozone at the C_9-10_ double bond with the net addition of two atoms of oxygen, and the remainder of the molecule appears to have been left unaltered. The oxidation state at C_8_ had an effect on the ratio of ozone required to form the intermediate products as well as result in degradation. In addition, from the report researched by Dellafiora [30], the changes of steric hindrances in the position 3 of DON may cause the loss of ribotoxicity under a structure-activity relationship perspective. From the possible structure of ozonation products of DON, the C_9-10_ double bond were opened of all six key degradation products, de-epoxy reaction were occurred in products A, B, D, and F, besides, steric hindrances in the position 3 was changed due to the broke of hydroxyl in products A, B, and D. According to the quantitative structure-activity relationship, the toxicity of ozonation products was significantly reduced compared with that of DON.

## 3. Conclusions

Gaseous ozone has significant effect on degradation of DON in ultrapure water, and six key degradation products of DON were deduced by UHPLC Q-Orbitrap-MS. In addition, the toxicity of the degradation products was also evaluated based on the structure-activity relationship. The TIC of degradation products showed that the ozonation products of DON in ultrapure water were complex in species and produce low concentrations. However, by using the UHPLC-Q-Orbitrap-MS technology, unknown ozonation products could be successfully screened and the structure were also be concluded. According to the structure of products, the addition reaction occurred in C_9-10_ double bond of all six ozonation products, de-epoxy reaction occurred and the changes of steric hindrances in the position 3 with the formation of most ozonation products, their toxicities were significantly reduced compared with those of DON. The preliminary toxicity evaluation of degradation products could provide important information for further animal experiment that is necessary to evaluate the safety of ozonation products of DON in wheat and other cereals. 

## 4. Materials and Methods 

### 4.1. Samples and Reagents

DON (purity ≥ 99%, analytical grade) used as the standard, was purchased from Sigma-Aldrich (St. Louis, MO, USA), CAS: 51481-10-8. LC-MS grade acetonitrile and methanol were supplied by Thermo Fisher Scientific (Rockford, IL, USA). Ultrapure water (18.2 MΩ·cm, 25 °C) was obtained from a Millipore-Q Advantage A10 Reagent Water System (Millipore, Bedford, MA, USA) and pre-filtered through 0.22 μm filter. 

The positive ion calibration solution, which includes n-butylamine (*m*/*z* 74), caffeine (*m*/*z* 195), MRFA (*m*/*z* 524) and Ultramark 1621 (*m*/*z* 1022, 1122, 1222, 1322, 1422, 1522, 1622, 1722, 1822); the negative ion calibration solution, which includes sodium dodecyl sulfate (*m*/*z* 265), sodium taurocholate (*m*/*z* 514) and Ultramark 1621, were used to tune and calibrate the Q-Orbitrap (Thermo Fisher Scientific, Rockford, IL, USA). 

### 4.2. Ozonation of DON

Gaseous ozone was generated from a laboratory corona discharge ozone generator (COM-AD-01, Sino-German joint venture, Anshan Ansi Ross Environmental Protection Co., Ltd., Anshan, China) using oxygen which purity was 99.999%, the flow rate of ozone was 0.2–2.0 L min^−1^, the maximum concentration of ozone could reach 150 mg L^−1^. Ultraviolet ozone detector (JSA9-O3-UV, Shenzhen JiShunAn Technology Co., Ltd., Shenzhen, China) had a wide detection range from 1 to 200 mg L^−1^, and accuracy was ± 0.1 mg L^−1^. 

The working solutions were transferred into test tubes and capped, gaseous ozone was directly applied into solutions. For ozonation effect analysis, concentration of 0, 1, 2, 4, and 8 mg L^−1^ gaseous ozone were used to treat 2 μg mL^−1^ of DON solution for 15 s; For degradation products analysis, 100 μg mL^−1^, 50 μg mL^−1^ and 20 μg mL^−1^ of DON were degraded by 10, 20, and 40 mg L^−1^ gaseous ozone for 180 s, respectively.

### 4.3. UHPLC/APCI Q-Orbitrap Analysis

Qualitative and Quantitative analysis of DON and degradation products were performed by UHPLC/APCI Q-Orbitrap system consisted of an Ultimate 3000 LC pump and an Ultimate open autosampler coupled with a Q-Orbitrap mass spectrometer (Thermo Fisher Scientific, Bremen, Germany). 

Mobile phase A of UHPLC was 0.1% formic acid in water, and mobile phase B was acetonitrile. The UHPLC column utilized was a Syncronis C_18_ 100 mm × 2.1 mm, 1.9 μm column (Thermo Fisher Scientific, Bremen, Germany). Gradient profile and flow rates were shown in Table 5. Column oven temperature was set at 30 °C, and autosampler temperature was set at 8 °C. Injection volume was 5 μL, and total run time was 25 min. 

### 4.4. Data Analysis

All experiments were replicated three times, the statistical analysis was carried out by using the Statistical Product and Service Solutions (SPSS) version 19.0. The results were considered with significant difference only when the P value was less than or equal to 0.05. 

The original spectra data was collected and analyzed by Xcalibur 3.0 software, screening of degradation products was performed by SIEVE 2.0 software, and Mass Frontier 7.0 was used to speculate the degradation path, these software were supplied by Thermo Fisher Scientific.

The elucidation of ozonation products were as follow: (1) Chromatography mass spectrometry information of DON before and after the gaseous ozone treatment were collected by using UHPLC-Q-Orbitrap high resolution mass spectrometry, and MS/MS fragment ion information were collected at the same time, from the total ions chromatogram (TIC) to make the pre-judgment if the new degradation products generated after ozone treatment. (2) Imported the original data into software SIEVE 2.0, which used for differential expression analysis, set the analyzing parameters and the software would provide all the different products compared with DON. (3) Compared the data generated by SIEVE with original data and confirmed the retention time (RT), exact masses, the possible empirical formula (assigned with errors of <5 ppm mass accuracy) and degree of unsaturation of degradation products. (4) Analyzed the effect of ozone concentration and initial concentration of DON on the degradation products, the validation of the key ozonation products based on the retention time reproducibility. (5) Based on the structure of DON, degree of unsaturation, MS/MS fragment ion information, the stability of the functional groups and small molecule structural elucidation software for mass spectrometry Mass Frontier 7.0, the structure of ozonation products of DON could be preliminarily confirmed.

## Figures and Tables

**Table 1:** Effect of gaseous ozone on deoxynivalenol (DON) in ultrapure water.

Ozone Concentration (mg L^−1^)	Concentration of DON (μg mL^−1^)	Degradation Rate of DON (%)
0	1.98 ± 0.02	0
1	1.55 ± 0.06	21.94
2	1.03 ± 0.14	47.96
4	0.21 ± 0.04	89.56
8	0.09 ± 0.01	95.68

Results are presented as the mean ± standard deviation (*n* = 3).

**Table 2:** Mass accuracy measurement of ozonation products of DON using UHPLC-Q-Orbitrap MS.

Retention Time (min)	Elemental Composition	Experimental Mass (*m*/*z*)	Theoretical Mass (*m*/*z*)	Error (ppm)	Degree of Unsaturation
4.31	C_15_H_18_O_7_	311.11209	311.11253	−1.41	7
4.54	C_14_H_16_O_6_	281.10159	281.10196	−1.32	7
4.67	C_15_H_20_O_7_	313.12738	313.12818	−2.55	6
4.74	C_15_H_18_O_9_	325.09128^*^	325.09179^*^	−1.59	7
5.27	C_15_H_22_O_9_	329.12253^*^	329.12309^*^	−1.72	5
5.68	C_15_H_20_O_10_	343.10199^*^	343.10236^*^	−1.07	6
5.87	C_15_H_20_O_10_	343.10178^*^	343.10236^*^	−1.70	6
6.49	C_15_H_18_O_8_	327.10687	327.10744	−1.75	7
7.00	C_15_H_20_O_9_	327.10678^*^	327.10744^*^	−2.03	6
8.46	C_14_H_18_O_7_	281.10168^*^	281.10196^*^	−1.14	6

The experimental mass and theoretical mass in the Table 2 are the *m*/*z* of [M+H]^+^, which means ionized mass with the less of an electron than the neutral mass M+H, and the “*” on the top right is the *m*/*z* of [M+H-H_2_O]^+^, which means one H_2_O molecule was lost than [M+H]^+^.

**Table 3:** Retention time reproducibility of six key ozonation products of DON.

Serial Number	Exact Mass	Formula	Experimental Mass (*m*/*z*)	Theoretical Mass (*m*/*z*)	Retention Time Reproducibility
A	310.10526	C_15_H_18_O_7_	311.11209	311.11253	4.32 ± 0.02
B	342.09509	C_15_H_18_O_9_	325.09131^*^	325.09179^*^	4.73 ± 0.01
C	346.12639	C_15_H_22_O_9_	329.12253^*^	329.12309^*^	5.27 ± 0.01
D	360.10565	C_15_H_20_O_10_	343.10178^*^	343.10236^*^	5.88 ± 0.01
E	326.10017	C_15_H_18_O_8_	327.10690	327.10744	6.48 ± 0.01
F	344.11074	C_15_H_20_O_9_	327.10687^*^	327.10744^*^	7.01 ± 0.02

The experimental mass and theoretical mass in the Table 3 are the *m*/*z* of [M+H]^+^, which means ionized mass with the less of an electron than the neutral mass M+H, and the “*” on the top right is the *m*/*z* of [M+H-H_2_O]^+^, which means one H_2_O molecule was lost than [M+H]^+^.

**Table 4:** Accurate mass measurements of six key ozonation products using UHPLC-Q-Orbitrap in the MS/MS mode.

Retention Time (min)	Formula	Experimental Mass (*m*/*z*)	Theoretical Mass (*m*/*z*)	Error (ppm)	Loss Formula
4.32	C_15_H_18_O_7_	311.11206	311.11253	−1.51	
	C_15_H_16_O_6_	293.10150	293.10196	−1.58	H_2_O
	C_14_H_16_O_5_	265.10663	265.10705	−1.59	CH_2_O_2_
	C_12_H_12_O_4_	221.08052	221.08084	−1.42	C_3_H_6_O_3_
	C_12_H_10_O_3_	203.06995	203.07027	−1.60	C_3_H_8_O_4_
	C_11_H_10_O_3_	191.06978	191.07027	−2.58	C_4_H_8_O_4_
4.73	C_15_H_18_O_9_	325.09134^*^	325.09179^*^	−1.40	
	C_14_H_16_O_8_	295.08102^*^	295.08123^*^	−0.70	CH_2_O
	C_13_H_14_O_7_	265.07022^*^	265.07066^*^	−1.67	C_2_H_4_O_2_
	C_12_H_12_O_6_	235.05975^*^	235.06010^*^	−1.47	C_3_H_6_O_3_
	C_11_H_12_O_5_	207.06506^*^	207.06519^*^	−0.59	C_4_H_6_O_4_
	C_8_H_10_O_6_	185.04431^*^	185.04445^*^	−0.75	C_7_H_8_O_3_
5.27	C_15_H_22_O_9_	329.12262^*^	329.12309^*^	−1.44	
	C_15_H_20_O_8_	311.11206^*^	311.11253^*^	−1.51	H_2_O
	C_14_H_20_O_7_	283.11719^*^	283.11761^*^	−1.51	CH_2_O
	C_14_H_18_O_6_	265.10675^*^	265.10705^*^	−1.13	CH_4_O_3_
	C_12_H_14_O_6_	237.07533^*^	237.07575^*^	−1.76	C_3_H_8_O_3_
	C_10_H_14_O_4_	181.08572^*^	181.08592^*^	−1.09	C_5_H_8_O_5_
5.88	C_15_H_20_O_10_	343.10208^*^	343.10236^*^	−0.81	
	C_15_H_18_O_9_	325.09167^*^	325.09179^*^	−0.37	H_2_O
	C_14_H_18_O_9_	313.09113^*^	313.09179^*^	−2.13	CH_2_O
	C_14_H_16_O_8_	295.08063^*^	295.08123^*^	−2.04	CH_4_O_2_
	C_13_H_16_O_8_	283.08102^*^	283.08123^*^	−0.72	C_2_H_4_O_2_
	C_13_H_16_O_7_	267.08588^*^	267.08631^*^	−1.64	C_2_H_4_O_3_
6.48	C_15_H_18_O_8_	327.10693	327.10744	−1.56	
	C_14_H_18_O_7_	299.11240	299.11253	−0.45	CO
	C_14_H_16_O_6_	281.10162	281.10196	−1.21	CH_2_O_2_
	C_13_H_14_O_6_	267.08606	267.08631	−0.96	C_2_H_4_O_2_
	C_12_H_12_O_4_	221.08058	221.08084	−1.15	C_3_H_6_O_4_
	C_11_H_12_O_4_	209.08034	209.08084	−2.38	C_4_H_6_O_4_
7.01	C_15_H_20_O_9_	327.10706^*^	327.10744^*^	−1.19	
	C_14_H_20_O_8_	299.11215^*^	299.11253^*^	−1.26	CO
	C_14_H_18_O_7_	281.10162^*^	281.10196^*^	−1.21	CH_2_O_2_
	C_13_H_16_O_7_	267.08728^*^	267.08631^*^	3.62	C_2_H_4_O_2_
	C_13_H_14_O_6_	249.07561^*^	249.07575^*^	−0.57	C_2_H_6_O_3_
	C_11_H_14_O_5_	209.08031^*^	209.08084^*^	−2.53	C_4_H_6_O_4_

The experimental mass and theoretical mass in the Table 4 are the *m*/*z* of [M+H]^+^, which means ionized mass with the less of an electron than the neutral mass M+H, and the “*” on the top right is the *m*/*z* of [M+H-H_2_O]^+^, which means one H_2_O molecule was lost than [M+H]^+^.

**Table 5:** Ultrahigh-Performance Liquid Chromatographic Gradient Profiles and MS Parameters.

**Gradient Profile and Flow Rate of UHPLC**			
Total Time (min)	Flow Rate (μL/min)	A (%)	B (%)
0	300	95	5
12	300	70	30
20	300	10	90
20.1	300	95	5
25	300	95	5
**Q-Exactive Parameters**			
sheath gas flow rate		35	
auxiliary gas flow rate		10	
sweep gas flow rate		0	
corona current (μA)		10	
capillary temperature (°C)		300	
s-lens level		55.0	
heater temperature (°C)		300	
scan range (*m*/*z*)		150–600	
resolution (± Full scan)		70,000	
resolution (dd-MS^2^)		17,500	
NCE		15	
